# Pridopidine selectively occupies sigma-1 rather than dopamine D2 receptors at behaviorally active doses

**DOI:** 10.1007/s00213-015-3997-8

**Published:** 2015-07-11

**Authors:** Kristoffer Sahlholm, Jurgen W. A. Sijbesma, Bram Maas, Chantal Kwizera, Daniel Marcellino, Nisha K. Ramakrishnan, Rudi A. J. O. Dierckx, Philip H. Elsinga, Aren van Waarde

**Affiliations:** Department of Nuclear Medicine and Molecular Imaging, University Medical Center Groningen, University of Groningen, Groningen, The Netherlands; Department of Physiology and Institute of Biomedical Technology (ITB), Center for Biomedical Research of the Canary Islands (CIBICAN), University of La Laguna School of Medicine, Tenerife, Spain; Department of Neuroscience, Karolinska Institutet, 171 77 Stockholm, Sweden

**Keywords:** MicroPET, Kinetic analysis, ^11^C-SA4503, ^11^C-raclopride, Receptor occupancy

## Abstract

**Rationale:**

Dopamine stabilizers have stimulatory actions under low dopamine tone and inhibitory actions under high dopamine tone without eliciting catalepsy. These compounds are dopamine D_2_ receptor (D_2_R) antagonists or weak partial agonists and may have pro-mnemonic and neuroprotective effects. The mechanism underlying their stimulatory and neuroprotective actions is unknown but could involve sigma-1R binding.

**Objectives:**

The present study examined sigma-1R and D_2_R occupancy by the dopamine stabilizer pridopidine (ACR16) at behaviorally relevant doses in living rats.

**Methods:**

Rats were administered 3 or 15 mg/kg pridopidine, or saline, before injection of the radiotracer ^11^C-SA4503 (sigma-1R) or ^11^C-raclopride (D_2_R). Some animals received 60 mg/kg pridopidine and were only scanned with ^11^C-raclopride. Cerebral ^11^C-SA4503 binding was quantified using metabolite-corrected plasma input data and distribution volume (*V*_T_) calculated by Logan graphical analysis. ^11^C-raclopride binding was quantified using striatum-to-cerebellum ratios and binding potentials calculated with a simplified reference tissue model.

**Results:**

Cunningham-Lassen plots indicated sigma-1R occupancies of 57 ± 2 and 85 ± 2 % after pretreatment of animals with 3 and 15 mg/kg pridopidine. A significant (44–66 %) reduction of ^11^C-raclopride binding was only observed at 60 mg/kg pridopidine.

**Conclusions:**

At doses shown to elicit neurochemical and behavioral effects, pridopidine occupied a large fraction of sigma-1Rs and a negligible fraction of D_2_Rs. Significant D_2_R occupancy was only observed at a dose 20-fold higher than was required for sigma-1R occupancy. The characteristics of dopamine stabilizers may result from the combination of high sigma-1R and low D_2_R affinity.

## Introduction

Pridopidine (also known as ACR16 or Huntexil) is a phenylpiperidine compound undergoing clinical trials for treatment of motor symptoms in Huntington’s disease (de Yebenes et al. 2011; Kieburtz et al. 2013; for the ongoing Pride-HD trial, see NCT02006472 at clinicaltrials.gov). Moreover, pridopidine has shown promising results in limited trials for schizophrenia and Parkinson’s disease (Ponten et al. 2010). The drug displays micromolar affinity for the dopamine D_2_ receptor (D_2_R), where it acts as an antagonist (Dyhring et al. 2010; Sahlholm et al. 2014) or very weak partial agonist (Seeman et al. 2009; Kara et al. 2010). Pridopidine antagonizes amphetamine-induced hyperlocomotion (Natesan et al. 2006; Ponten et al. 2010) and reduces L-DOPA-induced locomotor sensitization in 6-hydroxydopamine-lesioned rats (Ponten et al. 2013). However, even at doses resulting in near-complete D_2_R occupancy (assessed using ex vivo radioligand binding), pridopidine shows very low propensity for inducing catalepsy (Natesan et al. 2006). Furthermore, pridopidine stimulates locomotor activity in habituated animals showing low baseline locomotor activity (Rung et al. 2008) and increases dopamine release in several brain regions including the prefrontal cortex (Ponten et al. 2010). Based on these properties, pridopidine and structurally related compounds showing similar in vivo efficacy such as (−)-OSU6162 have been termed “dopamine stabilizers” as they exhibit inhibitory or stimulatory effects on dopamine-dependent behavior depending on the prevailing dopamine tone (Ponten et al. 2010).

(−)-OSU6162 displays higher in vitro affinity for D_2_R than pridopidine (Pettersson et al. 2010). Interestingly however, while (−)-OSU6162 was more potent in raising serum prolactin and in producing D_2_R occupancy, pridopidine was more potent in inhibiting amphetamine-induced hyperlocomotion (Natesan et al. 2006). Moreover, pridopidine showed pro-cognitive and pro-social effects, which are not typical of D_2_R ligands, in animal models of cognitive and negative symptoms of schizophrenia and in scopolamine-induced amnesia (Rung et al. 2005; Nilsson and Carlsson 2013). Furthermore, the abilities of haloperidol and (−)-OSU6162 to counteract amphetamine-induced hyperlocomotion were abolished in D_2_R knockout mice, whereas the effects of pridopidine persisted (Svensson et al. 2009). Based on the induction of the immediate-early gene Arc (a marker of synaptic activity) by pridopidine in prefrontal cortex, which again is not observed with other D_2_R antagonists or agonists, nondopaminergic effects of pridopidine have been postulated, which would involve an increase in cortical *N*-methyl-D-aspartate (NMDA) receptor activity (Ponten et al. 2010; Waters et al. 2014). Finally, neuroprotective effects of pridopidine and (−)-OSU6162 have recently been described in in vitro and in vivo models of Huntington’s disease (Ruiz et al. 2012; DiPardo et al. 2013). Taken together, the present literature findings suggest additional sites of action for pridopidine besides D_2_R, and as detailed below, we have considered the sigma-1 receptor (sigma-1R) as such an additional target.

The sigma-1R is a two-transmembrane protein that is widely expressed in the brain and has been implicated mainly in regulation of synaptic strength and cell survival. Sigma-1R lacks homology to any known mammalian protein, is believed to function both as an ER chaperone and as a co-receptor for a variety of other transmembrane proteins, and has been shown to modulate voltage-gated ion channels, NMDA receptors, and dopamine receptors (Maurice and Su 2009; Navarro et al. 2010; van Waarde et al. 2011). The sigma-1R is known to bind the endogenous steroids dehydroepiandrosterone-sulfate and progesterone and the hallucinogenic trace amine, *N*,*N*-dimethyltryptamine, along with exogenous substances such as cocaine and haloperidol (Fontanilla et al. 2009). On basis of the actions of its ligands in animal models, sigma-1R is considered as a pharmacological target for the treatment of Parkinson’s disease (including L-DOPA-induced dyskinesia), Alzheimer’s disease, schizophrenia, and drug addiction (Guitart et al. 2004; Maurice and Su 2009; van Waarde et al. 2011).

In radioligand competition experiments using ^3^H-(+)-pentazocine, pridopidine and (−)-OSU6162 were recently found to display nanomolar affinity for the sigma-1R (Sahlholm et al. 2013). For pridopidine, the observed affinity for sigma-1R was about 100-fold higher than that reported for D_2_R (high-affinity *K*_i_ about 70 nM at sigma-1R and 7520 nM at D_2_R), while (−)-OSU6162 showed only about two times higher affinity for sigma-1R than for D_2_R (high-affinity *K*_i_ about 360 nM at sigma-1R and 760 nM at D_2_R; Sahlholm et al. 2013; Pettersson et al. 2010). As detailed above, the effects of some sigma-1R ligands are similar to the actions of dopamine stabilizers that cannot easily be attributed to D_2_R antagonism. Therefore, we examined the occupancies of sigma-1R and D_2_R by pridopidine in the living rat brain. Pridopidine was administered at the lower end of the active dose range, as determined in previous behavioral and neurochemical studies, in order to assess a putative selective occupancy of sigma-1R at these doses.

## Materials and methods

### Radioligands and drugs

The D_2_R ligand ^11^C-raclopride was prepared by ^11^C-methylation of its hydroxy precursor (Farde et al. 1988). The tracer was dissolved in sterile saline. Specific radioactivity was >10 GBq/μmol at the time of injection. The sigma-R ligand 1-[2-(3,4-dimethoxyphenethyl)]-4-(3-phenylpropyl)piperazine (^11^C-SA4503) was produced by ^11^C-methylation of 4-O-demethyl SA4503 (Kawamura et al. 2003). The isotonic tracer solution had a pH of 6.0 to 7.0, a specific radioactivity >15 GBq/μmol at the moment of injection, and a radiochemical purity >98 %. Pridopidine hydrochloride (ACR16) was custom synthesized by Axon MedChem BV (Groningen, Netherlands). HPLC grade ethanol was from Merck (Darmstadt, Germany), and methanol and acetonitrile were purchased from Rathburn (Walkerburn, Scotland).

### Animals

Male Wistar Unilever rats were obtained from Harlan (Boxmeer, Netherlands). The animals were housed in Makrolon cages on a layer of wood shavings at constant temperature (21 ± 2 °C) and a 12-to-12 h light-dark regimen. They were fed standard laboratory chow (RMH-B, Hope Farms, The Netherlands) and received food and water ad libitum. The rats were allowed to acclimate for at least 7 days after they had been transported to Groningen. The research protocol was approved by the Institutional Animal Care and Use Committee of Groningen University (file no. 6867A). All experiments were performed by licensed investigators in compliance with the Law on Animal Experiments of The Netherlands.

### Drug dosing and blood sampling

Saline or pridopidine dissolved in saline (3, 15, or 60 mg/kg, s.c.) was administered to awake rats 60 min before tracer injection. Data of the animals (body weight, injected tracer dose, number of subjects in each dose group) are listed in Table 1. Animals were first scanned with ^11^C-raclopride and subsequently with ^11^C-SA4503, after an interval of at least 1 week, with exception of the animals that received 60 mg/kg pridopidine. Because of limited availability of the test drug, the 60 mg/kg dose group was scanned only with ^11^C-raclopride.

**Table 1:** Data concerning the experimental animals (body weights, injected doses, group sizes)

Data concerning ^11^C-raclopride scans			
Pridopidine dose (mg kg)	Body weight at scan date (g)	^11^C-raclopride injected (MBq)	Number of animals (*N*)
0	315 ± 5	37 ± 4	4
3	339 ± 19	23 ± 5	5
15	316 ± 10	22 ± 5	4
60	350 ± 9	25 ± 1	2
Data concerning ^11^C-SA4503 scans			
Pridopidine dose (mg kg)	Body weight at scan date (g)	^11^C-SA4503 injected (MBq)	Number of animals (*N*)
0	354 ± 28	15 ± 5	4
3	372 ± 23	13 ± 3	4
15	346 ± 10	11 ± 4	4
Data concerning ^11^C-SA4503 metabolite assays			
Pridopidine dose (mg kg)	Body weight at scan date (g)	^11^C-SA4503 injected (MBq)	Number of animals (*N*)
0	301 ± 17	24 ± 3	2
3	366 ± 79	33 ± 6	2
15	336 ± 28	23 ± 4	2

Body weights and injected doses are listed as mean ± SEM

After drug administration and about 20 min before each microPET scan, animals were anesthetized with isoflurane (Pharmachemie BV, Haarlem, Netherlands). A tail vein cannula was placed in each rat for injection of ^11^C-raclopride and a femoral vein cannula for injection of ^11^C-SA4503. In the case of ^11^C-SA4503 scans, a femoral artery cannula was also placed for blood sampling and determination of the time course of radioactivity in plasma, as described previously (Ramakrishnan et al. 2014). Both tracers were injected as a slow bolus (1 ml of saline-diluted tracer solution infused during a period of 1 min), using a Harvard-style injection pump. By the use of a pump, inter-individual variability in the outcome parameters of kinetic modeling is reduced (Visser et al. 2013).

From each animal used for kinetic modeling (Table 1), 15 arterial blood samples (volume 0.1 to 0.15 ml) were drawn at 0.17, 0.33, 0.5, 0.67, 0.83, 1, 1.5, 2, 3, 5, 7.5, 10, 15, 30, and 60 min after ^11^C-SA4503 injection and the start of the microPET scan. From these samples, 25 μl of whole blood was collected and plasma (25 μl) was then obtained by centrifugation of the remaining sample (5 min at 13,000*×g*). Radioactivity in plasma and whole blood was determined using a calibrated gamma counter (CompuGamma CS1282, LKB-Wallac, Turku, Finland). Results are expressed as standardized uptake values (SUV), defined as [(plasma activity concentration (MBq/g) × body weight (g) / injected dose (MBq)]. Rat plasma was assumed to have a specific gravity of 1 g/ml.

Separate animals were used for analysis of tracer metabolites in plasma. In these rats, five arterial blood samples were collected at 5, 10, 20, 40, and 60 min. Plasma was obtained from these samples by centrifugation (2 min at 13,000*×g*). One volume of plasma was mixed with one volume of a 1:1 mixture of 20 % trichloroacetic acid and acetonitrile. Proteins were precipitated by centrifugation (2 min at 13,000*×g*). The clear supernatant was injected in a reversed-phase HPLC system to separate parent tracer and metabolites. An Alltima C18 column (250 × 10 mm, 5 μ) was used; the mobile phase consisted of 100 mM NaH_2_PO_4_/ethanol 55:45 (*v*/*v*) at a flow rate of 3 ml/min. The eluate was collected in 20-s fractions for 12 min, and radioactivity in the fractions was measured using a gamma counter. Retention time of authentic ^11^C-SA4503 was 7.5 min whereas two metabolites were eluted at retention times of 4.3 and 6.3 min. The areas under the radioactive peaks were calculated, and results were expressed as the fraction of plasma radioactivity representing parent tracer (in %).

### MicroPET scans

Two rats were scanned in each scan session. They were positioned on heating mats in a microPET Focus 220 system, in transaxial position above each other with their brains in the field of view. Body temperature of the animals was kept close to normal with electronic temperature controllers. Heart rate and oxygen level of the blood during the scan were monitored using pulse oximeters (PulseSense, Nonin). A transmission scan (duration 515 s) with a Co-57 point source was first made, in order to correct the subsequently acquired emission data for attenuation and scatter of 511 keV photons. The first rat was injected with tracer (^11^C-raclopride or ^11^C-SA4503) simultaneously with the start of the acquisition of emission data by the PET camera, whereas the second animal was injected 16 min later. A list mode protocol was used with a 76-min acquisition time.

Image reconstruction was performed using microPET Manager 2.3.3.6 (Siemens). The PET data were split according to position along the *y*-axis, so that a separate data set was acquired for each animal. The list mode data of the emission scans were reframed in a dynamic sequence of 6 × 10, 4 × 30, 2 × 60, 1 × 120, 1 × 180, 4 × 300, and 3 × 600 s (with an additional 960-s frame for the rat that was injected first). The data were reconstructed per time frame employing an iterative reconstruction algorithm (OSEM2D with Fourier rebinning, 4 iterations, and 16 subsets). The final datasets consisted of 95 slices with a slice thickness of 0.8 mm and an in-plane image matrix of 128 × 128 pixels. Data sets were corrected for decay, random coincidences, scatter, and attenuation.

### Data analysis

**Fig. 1:**
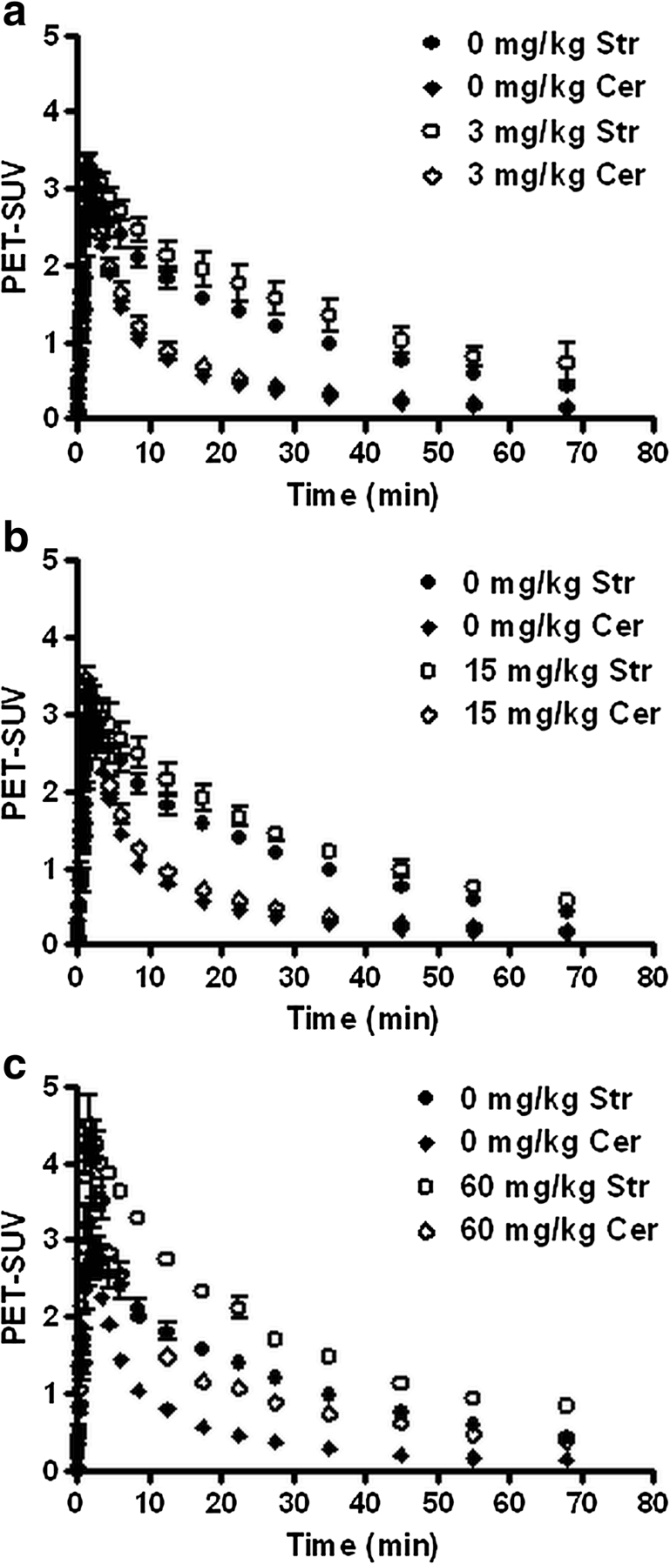
Time-activity curves of the dopamine D_2_ receptor ligand ^11^C-raclopride in rat striatum (*Str*) and cerebellum (*Cer*) after treatment of animals with 3 (**a**), 15 (**b**), or 60 mg/kg pridopidine (**c**). Data are plotted as mean ± SEM

In ^11^C-raclopride scans of the brain, three-dimensional regions of interest (ROIs) were manually drawn over the left and right striatum and cerebellum, using the program ASIPro 6.3.3.0 (Siemens). Each ROI had a standard size of 50 μl. Time-activity curves (TACs) were generated for these regions (Fig. 1). The results are expressed as dimensionless standardized uptake values (PET-SUV), defined as [(tissue activity concentration (MBq/g) × body weight (g) / injected dose (MBq)]. Brain tissue was assumed to have a specific gravity of 1 g/ml.

**Fig. 2:**
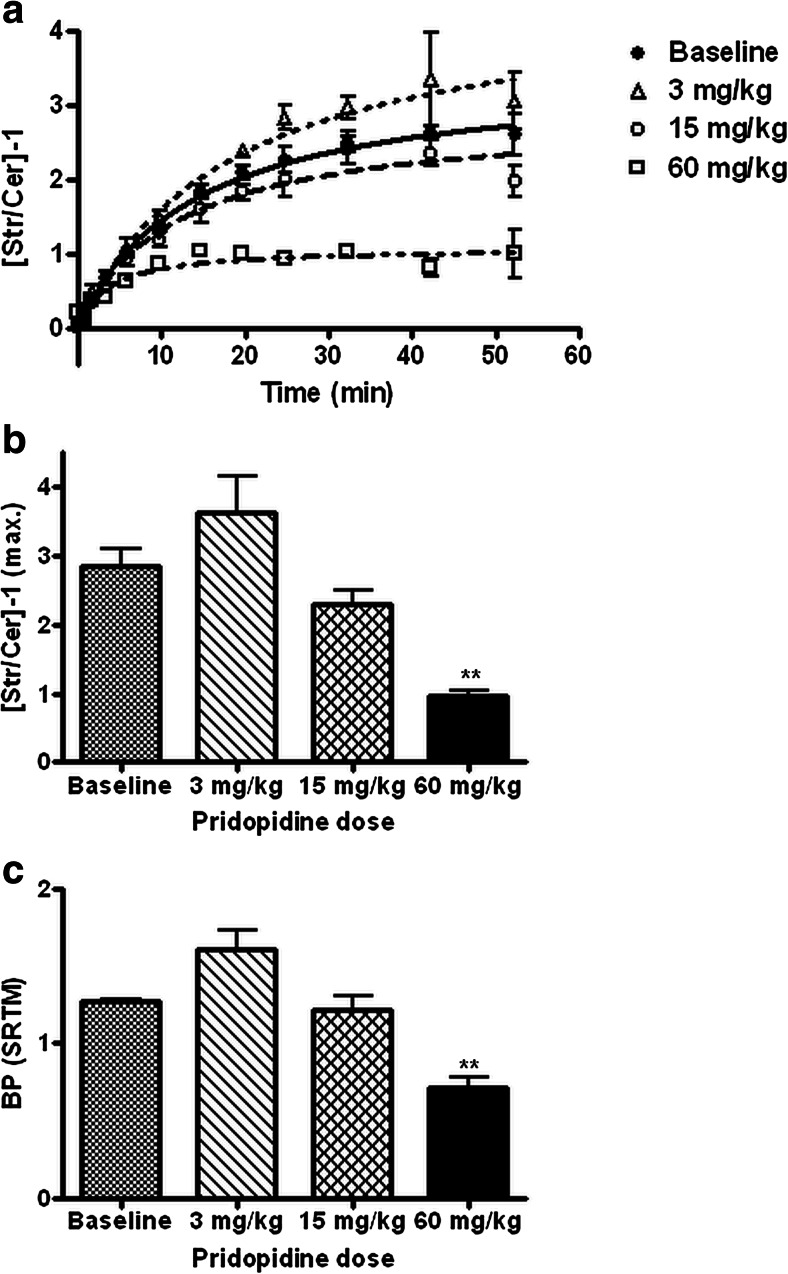
Time-dependent striatum-to-cerebellum ratios of radioactivity (**a**), plateau levels of these ratios minus one (**b**), and binding potential values for ^11^C-raclopride in rat striatum calculated with a simplified reference tissue model (**c**). Data are plotted as mean ± SEM

Time-dependent striatum-to-cerebellum ratios of radioactivity were calculated from these TACs, and a first-order rate equation was fitted to these data (Fig. 2a). Plateau values for the striatum-to-cerebellum ratio were derived from this curve fit. A simplified reference tissue model (Lammertsma and Hume 1996) was also fitted to the striatal TACs, using cerebellum as reference.

PET images for ^11^C-SA4503 were co-registered with an MRI template using the program PMOD (PMOD Technologies Ltd, Zürich, Switzerland), and three-dimensional ROIs for individual brain regions were copied from MRI to PET as described previously (Ramakrishnan et al. 2014). Logan graphical analysis was used to calculate regional tracer distribution volumes (*V*_T_). The starting time of the Logan fit was 20 min. Metabolite-corrected plasma radioactivity data were used as input function, and (uncorrected) whole blood radioactivity served to estimate the contribution of blood to brain radioactivity measured by the PET camera. The plasma time-activity curve (TAC) of each animal was corrected for metabolites using an exponential function obtained from the average metabolite curve of rats which had received the same dose of pridopidine. The PMOD software package was used for curve fitting. A Cunningham-Lassen plot (Lassen et al. 1995; Cunningham et al. 2010) was made to estimate receptor occupancy, as described previously (Ramakrishnan et al. 2014).

### Statistics

Differences between AUCs were examined using the *t* test. The biodistribution, *V*_T_ and *BP* data were analyzed using ANOVA. A post hoc Bonferroni test was done when applicable. *P* values <0.05 were considered statistically significant.

## Results

### ^11^C-raclopride kinetics in brain

The D_2_R ligand ^11^C-raclopride entered the brain within 3 min and was washed out more rapidly from the reference region (cerebellum) than the target region (striatum) (Fig. 1a). Pretreatment of animals with pridopidine had a dose-dependent effect on the ^11^C-raclopride time-activity curves. At the lowest dose tested (3 mg/kg), pridopidine appeared to increase tracer uptake in striatum but not in cerebellum (Fig. 1a). When the pridopidine dose was raised to 15 mg/kg, the drug appeared to increase tracer uptake in both striatum and cerebellum to a rather minor extent (Fig. 1b). Administration of 60 mg/kg pridopidine resulted in a striking increase of the uptake of ^11^C-raclopride in both striatum and cerebellum (Fig. 1c). The relative increase in striatum (compared to uptake at baseline) was smaller than the relative increase in cerebellum.

### Target-to-nontarget ratios of ^11^C-raclopride

In the brain of animals scanned with ^11^C-raclopride, striatum-to-cerebellum ratios of radioactivity rose to a plateau value which was reached after a time interval ranging from 15 to 60 min, depending on the administered pridopidine dose (Fig. 2a). The level of this plateau could be estimated by fitting a first-order rate equation to the PET data. After administration of a low dose of pridopidine (3 mg/kg), the plateau value appeared to be increased (Fig. 2). A higher pridopidine dose (15 mg/kg) produced a slight decrease of the level of the plateau (Fig. 2). Particularly, the lowest dose of pridopidine increased the inter-individual variability in the kinetics of ^11^C-raclopride in striata; for this reason, neither the effect of 3 mg/kg nor that of 15 mg/kg pridopidine on the level of the plateau was statistically significant (Fig. 2). Administration of 60 mg/kg pridopidine resulted in a significant decrease of the target-to-nontarget ratio of ^11^C-raclopride (Fig. 2). Target binding of the radioligand (striatum-to-cerebellum ratio at plateau minus one) was reduced from 2.85 ± 0.48 at baseline to 0.96 ± 0.10 by 60 mg/kg pridopidine (mean ± S.D., *P* < 0.01, Fig. 2b), corresponding to a D_2_R occupancy of 66 %.

A simplified reference tissue model (SRTM) could be fitted to the PET data of ^11^C-raclopride, using striatum as the target region and cerebellum as the reference region. Binding potential values calculated with the SRTM demonstrated similar drug-induced changes as striatum-to-cerebellum ratios (Fig. 2c). However, D_2_R occupancy by pridopidine at 60 mg/kg calculated from the SRTM fit was lower than values calculated using the striatum-to-cerebellum ratio (44 vs. 66 %, respectively; see Figs. 2c, b).

### ^11^C-SA4503 kinetics in brain and plasma

**Fig. 3:**
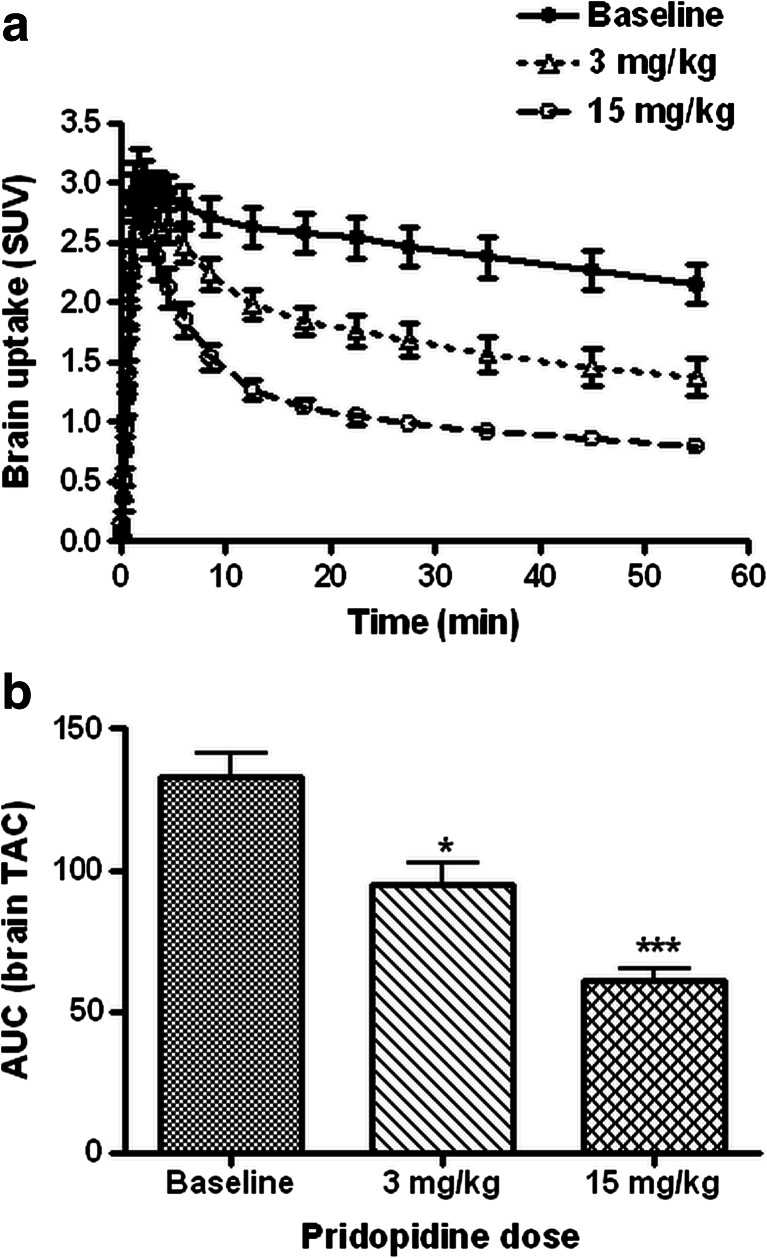
Time-activity curves of the sigma-1 receptor ligand ^11^C-SA4503 in the entire rat brain (**a**) and areas under the cerebral time-activity activity curve (**b**). Data are plotted as mean ± SEM

Uptake of the sigma-1 receptor ligand ^11^C-SA4503 in the brain was rapid (Fig. 3a). A maximum was reached within the first 5 min after tracer injection and was followed by washout. The rate of this washout was increased after pridopidine treatment, and cerebral binding of the ligand was dose-dependently reduced (Fig. 3a). The area under the brain time-activity curve (AUC) was significantly decreased by pridopidine, at both 3 and 15 mg/kg (*P* <0.02 and <0.0005, respectively, Fig. 3b).

**Fig. 4:**
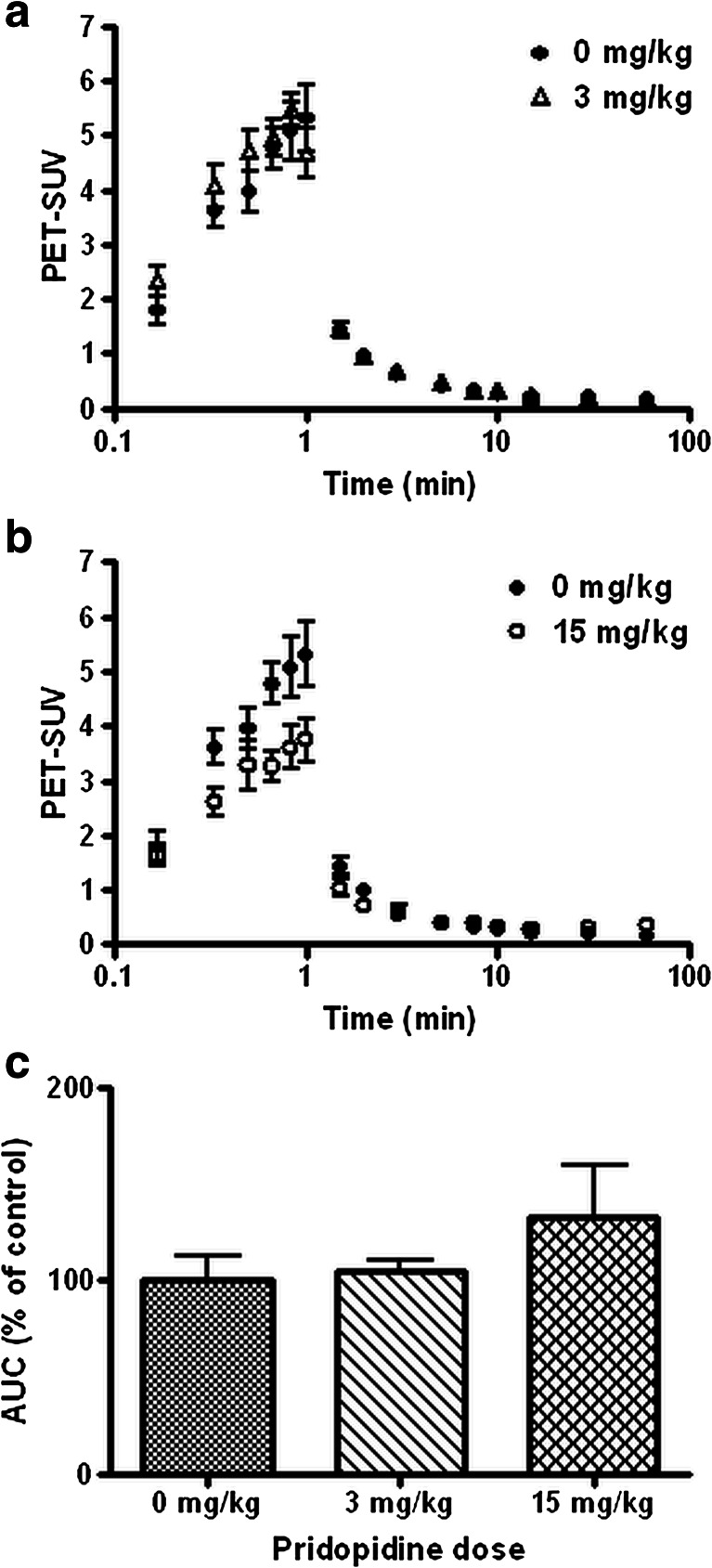
Time-activity curves of radioactivity in rat plasma after injection of the sigma-1 receptor ligand ^11^C-SA4503 (**a**, **b**; note logarithmic *x*-axis) and areas under the plasma time-activity curve (**c**). Data are plotted as mean ± SEM

Pretreatment of animals with 3 mg/kg pridopidine had very little impact on the clearance of ^11^C-SA4503-derived radioactivity from plasma (Fig. 4a). However, after treatment of animals with 15 mg/kg pridopidine, the shape of the plasma curve was altered (Fig. 4b). Compared to untreated controls, plasma levels of radioactivity were initially reduced (during the first 5 min after tracer injection) but subsequently increased (between 10 and 60 min). SUV values in Fig. 4 are plotted on a logarithmic *x*-axis in order to avoid that all data points from the initial frames would overlap. Since AUC takes frame length into account, a small change of radioactivity in a late frame has more impact on the calculated area than a considerable change in an early frame. The area under the plasma curve tended to be increased after pridopidine treatment, by 5 and 33 % at doses of 3 and 15 mg/kg, respectively (Fig. 4c). However, neither of these changes were statistically significant.

### Impact of pridopidine on ^11^C-SA4503 metabolism

**Fig. 5:**
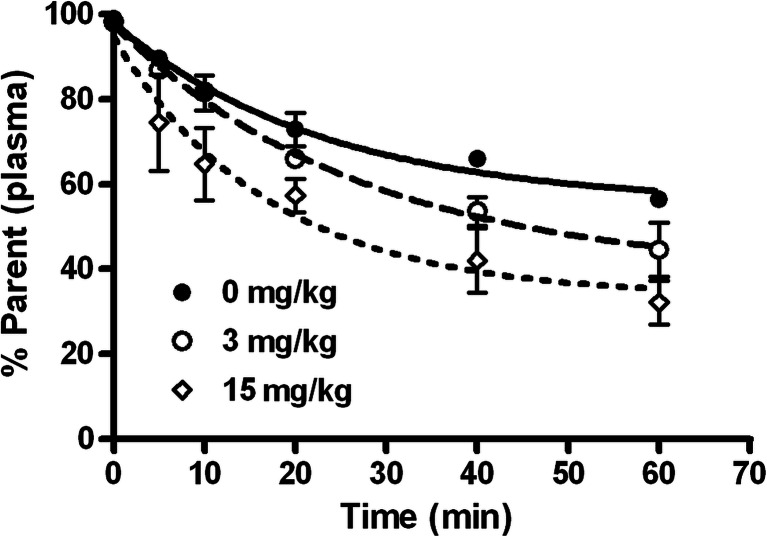
Fraction of plasma radioactivity representing intact parent compound after injection of ^11^C-SA4503. Each data point represents the mean value of two rats

Tracer metabolism was dose-dependently accelerated after treatment of rats with various doses of pridopidine (Fig. 5). While 55 % of the parent tracer remained unchanged at 60 min in saline-treated rats, only 39 and 33 % remained in the 3 and 15 mg/kg groups.

### Kinetic analysis and sigma-1 receptor occupancy

**Fig. 6:**
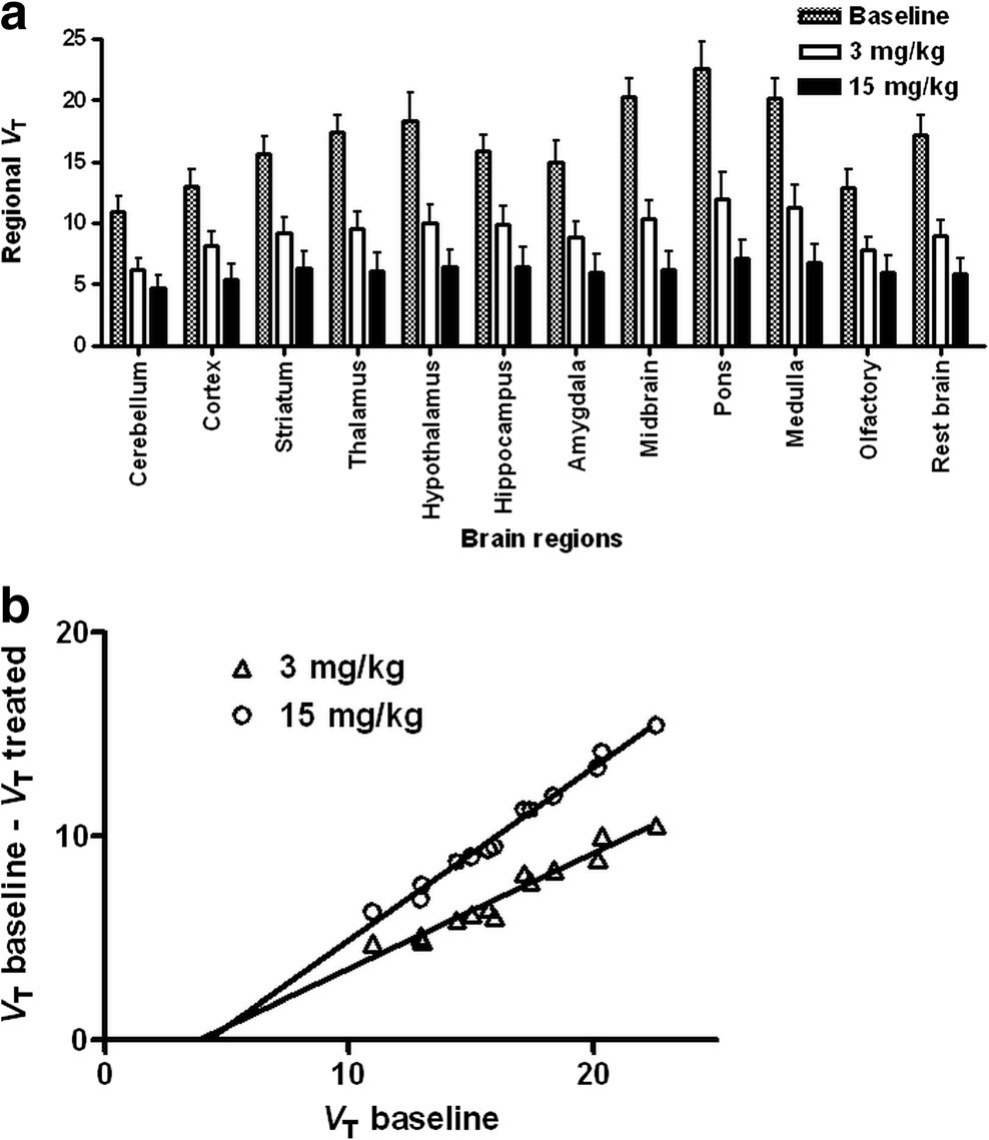
Total distribution volume (*V*_T_) of ^11^C-SA4503 in different brain regions estimated using Logan graphical analysis (**a**) and Cunningham-Lassen plot of sigma-1 receptor occupancy by pridopidine (**b**). Each data point in the plot represents an individual brain region

Logan graphical analysis was performed to estimate *V*_T_ in the whole brain and in different brain regions. Increasing doses of pridopidine produced a dose-dependent reduction in *V*_T_ for all regions investigated (Fig. 6a). The reduction was statistically significant at either dose. The Lassen plot as modified by Cunningham was used to estimate receptor occupancy and *V*_ND_ (Fig. 6b). Receptor occupancy was 56.7 ± 2.3 % at 3 mg/kg and 84.5 ± 1.7 % at 15 mg/kg. The *V*_ND_ calculated as an average of the two doses administered was 3.99. Since the average whole brain *V*_T_ (after metabolite correction) was 14.39, the specific binding fraction of ^11^C-SA4503 in rat brain was >72 %, very similar to the value of 75 % previously reported using the same method in Wistar-Hannover rats (Ramakrishnan et al. 2014).

### Biodistribution data

Biodistribution data for ^11^C-SA4503-derived radioactivity are listed in Table 2. Pridopidine reduced radioligand uptake in the brain, large intestine, and kidney. However, the plasma SUV was significantly increased in the 15 mg/kg dose group. Therefore, tissue-to-plasma ratios of radioactivity provide more information about the drug effect than raw SUV values. Dose-dependent reductions of these ratios were observed in all studied areas of the brain, the kidney, and the spleen. In some other tissues (adrenal gland, bone marrow, large and small intestine, lung), reductions were noted only after administration of the high (15 mg/kg) dose of pridopidine. Drug effects on tissue-to-plasma ratios in liver and pituitary gland did not reach statistical significance.

**Table 2:** Biodistribution data of ^11^C-SA4503 in the various treatment groups

Tissue	Baseline	3 mg/kg	15 mg/kg
Uptake of radioactivity (SUV values), 80 min after injection of ^11^C-SA4503			
Cerebellum	1.95 ± 0.12	0.76 ± 0.17***	0.73 ± 0.03***
Cerebral cortex	1.97 ± 0.15	1.17 ± 0.17*	0.87 ± 0.05**
Rest brain	2.23 ± 0.31	1.10 ± 0.05*	0.65 ± 0.03*
Adipose tissue	0.26 ± 0.05	0.27 ± 0.06	0.36 ± 0.04
Adrenal gland	25.7 ± 3.6	23.0 ± 4.1	10.7 ± 4.1
Bladder	0.70 ± 0.10	0.88 ± 0.38	0.83 ± 0.22
Bone	0.44 ± 0.14	0.26 ± 0.03	0.44 ± 0.05
Bone marrow	2.57 ± 0.47	2.62 ± 0.67	2.20 ± 0.77
Heart	0.33 ± 0.04	0.23 ± 0.01	0.38 ± 0.02
Intestine (large)	1.72 ± 0.13	1.21 ± 0.12*	0.74 ± 0.08***
Intestine (small)	2.33 ± 0.28	1.93 ± 0.40	1.70 ± 0.15
Kidney	4.19 ± 0.20	2.10 ± 0.09***	2.07 ± 0.15***
Liver	6.47 ± 0.63	7.71 ± 0.89	7.21 ± 0.83
Lung	1.98 ± 0.18	1.05 ± 0.06*	1.47 ± 0.04
Muscle	0.15 ± 0.02	0.13 ± 0.01	0.22 ± 0.02*
Pancreas	4.95 ± 0.74	5.02 ± 0.64	6.23 ± 0.53
Pituitary	3.24 ± 0.03	2.13 ± 0.44	2.75 ± 0.72
Plasma	0.11 ± 0.02	0.14 ± 0.02	0.28 ± 0.02***
Red blood cells	0.10 ± 0.03	0.07 ± 0.01	0.16 ± 0.01
Spleen	2.62 ± 0.27	2.03 ± 0.12	1.99 ± 0.07
Submandibular gland	3.60 ± 0.18	4.48 ± 0.64	5.77 ± 0.39
Urine	0.35 ± 0.16	1.60 ± 0.87	0.50 ± 0.09
Tissue-to-plasma ratios of radioactivity, 80 min after injection of ^11^C-SA4503			
Cerebellum	20.0 ± 2.8	5.9 ± 1.5**	2.7 ± 0.3**
Cerebral cortex	20.1 ± 2.9	9.0 ± 1.7*	3.2 ± 0.3**
Rest brain	22.0 ± 2.1	8.4 ± 1.3***	2.4 ± 0.2***
Adipose tissue	2.6 ± 0.5	1.8 ± 0.2	1.3 ± 0.1
Adrenal gland	186 ± 13	184 ± 49	38 ± 14***
Bladder	7.7 ± 2.0	6.9 ± 3.4	3.2 ± 1.0
Bone	4.0 ± 0.8	2.1 ± 0.4	1.6 ± 0.2
Bone marrow	20.6 ± 2.6	21.3 ± 8.1	8.6 ± 3.4*
Heart	3.4 ± 0.7	1.7 ± 0.2	1.4 ± 0.1
Intestine (large)	18.5 ± 4.2	9.5 ± 2.1	2.8 ± 0.4*
Intestine (small)	25.4 ± 6.1	13.6 ± 1.6	6.2 ± 0.3*
Kidney	44.3 ± 8.7	16.1 ± 2.3*	7.6 ± 0.3*
Liver	71.6 ± 18.7	59.3 ± 12.4	27.2 ± 4.7
Lung	21.0 ± 4.7	8.3 ± 1.6	5.5 ± 0.5*
Muscle	1.7 ± 0.4	1.0 ± 0.2	0.8 ± 0.1
Pancreas	52.2 ± 11.5	36.7 ± 2.5	23.2 ± 2.7
Pituitary	23.6 ± 0.5	16.7 ± 4.6	10.7 ± 3.4
Red blood cells	0.8 ± 0.1	0.5 ± 0.1*	0.6 ± 0.0*
Spleen	26.3 ± 2.6	16.0 ± 3.2*	7.4 ± 0.7**
Submandibular gland	38.1 ± 7.6	32.3 ± 0.5	21.6 ± 2.6
Urine	4.6 ± 2.4	13.5 ± 7.8	1.9 ± 0.4

Data are listed as mean ± SEM. *N* = 4 in all groups

*P < 0.05, **P < 0.01, ***P < 0.005 (compared to baseline value)

## Discussion

The 3 and 15 mg/kg subcutaneous pridopidine doses used in the present study were chosen since they are at the lower end of the dose range known to produce neurochemical and behavioral effects in rats, e.g., 1.7 mg/kg increased dopamine release in striatum (Ponten et al. 2010), 7.9 mg/kg decreased L-DOPA-induced rotational asymmetry in 6-hydroxydopamine-hemilesioned rats (Ponten et al. 2013), 10 mg/kg induced Arc gene expression in frontal cortex (Waters et al. 2014), and 15.9 mg/kg decreased MK-801- and amphetamine-induced hyperlocomotion and increased locomotor activity in habituated rats (Rung et al. 2008; Ponten et al. 2010).

No significant changes in ^11^C-raclopride binding were observed following treatment with 3 and 15 mg/kg pridopidine. Curiously however, there was a trend for increased ^11^C-raclopride binding at 3 mg/kg, along with an increase in the variability of radiotracer binding between animals. The mechanisms behind these effects are unclear but could potentially involve sigma-1R-mediated alterations of D_2_R expression or binding properties, since D_2_R and sigma-1R have been shown to form heteromers in striatum (Navarro et al. 2013). Based on published ex vivo data (Natesan et al. 2006), we expected to detect substantial D_2_R occupancy at 15 mg/kg. Since we did not observe a significant decrease in ^11^C-raclopride binding at this dose, we evaluated 60 mg/kg in two additional animals to confirm D_2_R binding by pridopidine using in vivo PET. Indeed, at this elevated dose, a D_2_R occupancy of 66 or 44 % (as determined using the striatum-to-cerebellum ratio or SRTM methods, respectively) was observed. Binding potential (BP) data calculated from the SRTM fit are probably the most reliable, since data from the ratio method can deviate from the true BP values, depending on the kinetics of tracer clearance from plasma and tissue. Our SRTM data correspond closely to BP values reported earlier by Lammertsma and Hume (1996). Particularly at 60 mg/kg, pridopidine seemed to increase the levels of ^11^C-raclopride in rat plasma, resulting in increased nondisplaceable binding of the tracer throughout the brain (Fig. 1c).

The results of the present study are somewhat divergent from those of Natesan et al. 2006, who observed substantial D_2_R occupancy (35 %) at 10 mg/kg and an ED_50_ of 18.99 mg/kg. The use of different methodology (in vivo PET vs. ex vivo radioligand binding) and/or different strains of rats (Wistar Unilever vs. Sprague-Dawley) may partially explain the differences in the results obtained. Moreover, isoflurane anaesthesia, which may inhibit dopamine release (Westphalen et al. 2013), was used during in vivo PET recordings but not in the previous study. Therefore, the larger pridopidine-induced decreases in ^3^H-raclopride binding observed by Natesan et al. (2006) may have been produced both by increases of dopamine release and by occupancy of D_2_R by pridopidine. However, other researchers have reported that the sensitivity of ^3^H-raclopride binding to amphetamine-induced dopamine release was not significantly affected by isoflurane (McCormick et al. 2011).

Pridopidine was administered s.c. since this route of administration was used in the occupancy study by Natesan et al. (2006), as well as in the majority of behavioral studies cited in the present work. It could be argued that pridopidine uptake in brain may be submaximal 1 h after s.c. administration, due to slow redistribution from the injection site, resulting in underestimation of D_2_R occupancy. However, time course data reported by Natesan et al. (2006) suggest that maximal D_2_R occupancy is reached already within 1 h after s.c. injection and is maintained for 4 h. It could also be argued that different radiotracer affinities may have biased the present results. The affinities of ^11^C-raclopride and ^11^C-SA4503 for their respective targets are not very different (*K*_d_s in rat brain tissue of 1.2 and 4.5 nM, respectively; Köhler et al. 1985; Ishiwata et al. 2003). Furthermore, at the tracer doses used in PET, radiotracer affinity should not affect occupancy measurements when the competitor (pridopidine in this case) is present at constant levels (Laruelle 2000). Considering the time course data cited above, the assumption of constant competitor levels seems reasonable.

Human occupancy data for pridopidine have not yet been reported, but a recent ^11^C-raclopride study (Tolboom et al. 2015) examined D_2_R occupancy by (−)-OSU6162 and observed a 2 to 33 % occupancy in human subjects that received doses ranging from 15 to 90 mg (between 0.14 and 1.25 mg/kg). Since the dose-occupancy curve appeared to level off at 40 %, the authors suggested that (−)-OSU6162 selectively occupies a subset of striatal D_2_Rs, a property which could potentially account for some of the characteristics of dopamine stabilizers. However, as cautioned by the authors, firm conclusions concerning maximal occupancy could not be drawn since a limited dose range was examined (for safety reasons) and since the number of subjects was small. Ekesbo et al. (1999) reported a 76 % displacement of ^11^C-raclopride by continuous infusion of (−)-OSU6162 (3 mg/kg/h) in rhesus monkeys, suggesting that in nonhuman primates, a large fraction of striatal D_2_Rs is accessible to (−)-OSU6162, similar to rodents (Natesan et al. 2006). Our microPET data suggest that D_2_Rs in rat brain are only occupied by pridopidine at relatively high (60 mg/kg) doses.

Dopamine stabilizers show some characteristics which are not usually associated with D_2_R ligands, such as pro-cognitive actions in natural forgetting and scopolamine-induced amnesia (Nilsson and Carlsson 2013), neuroprotective effects in in vitro and in vivo models of Huntington’s disease (Ruiz et al. 2012; DiPardo et al. 2013), and increased social interaction in the MK-801 rat model of social withdrawal in schizophrenia (Rung et al. 2005). In this context, the substantial occupancy of sigma-1R by pridopidine at 3 and 15 mg/kg, as demonstrated in the present study, is of particular interest since sigma-1R ligands have been shown to possess anti-amnesic and neuroprotective actions, efficacy against negative symptoms in schizophrenia, and positive modulatory effects on NMDA receptor activity (Maurice and Su 2009; Hayashi et al. 2011; van Waarde et al. 2011). If verified in a patient population, the potentially disease-modifying neuroprotective effects of pridopidine would be highly relevant for the treatment of Huntington’s patients. Sigma-1R has generated much attention as a target for neuroprotective treatment, and the sigma-1R agonist PRE-084 was recently demonstrated to alleviate motor impairments and restore dopamine terminal density in 6-hydroxydopamine-lesioned mice, an effect which was not observed in sigma-1R knockout animals (Francardo et al. 2014). Finally, sigma-1R ligands have been reported to attenuate the locomotor effects elicited by methamphetamine (Matsumoto et al. 2008), which could explain the findings reported by Natesan et al. (2006) and Svensson et al. (2009) that the ability of pridopidine to inhibit amphetamine-induced locomotion is more potent than that of (−)-OSU6162 and persists in D_2_R knockout mice.

In conclusion, the present study confirms a selective preference of pridopidine for sigma-1Rs over D_2_Rs and demonstrates that pridopidine, in vivo, occupies a large fraction of sigma-1Rs at the lower end of its behaviorally and neurochemically active dose range, while requiring higher doses to produce significant D_2_R occupancy. Our findings warrant further investigation of the role of sigma-1 receptors in the behavioral and neuroprotective effects of dopamine stabilizers and of pridopidine in particular. However, the pharmacological characterization of sigma-1R-mediated effects may prove challenging since the classification of sigma ligands into agonists and antagonists remains tentative. The functional effects of sigma-1R ligands in different cellular and subcellular contexts may even depend on the heteromeric partners of sigma-1Rs. Furthermore, whereas several prescribed CNS drugs are known to significantly occupy sigma-1R at therapeutic doses, the relevance of this occupancy for treatment response has not yet been convincingly demonstrated. Future experiments employing sigma-1R knockout animals, rather than a pharmacological blocking approach, may yield more easily interpretable data.
